# Health professionals’ perceptions, barriers and knowledge towards oral health care of dependent people in nursing homes: a systematic review

**DOI:** 10.3389/fpubh.2024.1504542

**Published:** 2025-01-23

**Authors:** Joana Pombo-Lopes, Inês Rodrigues, Joana Costa, Ana Catarina Gomes, Jorge Fonseca, José Grillo-Evangelista

**Affiliations:** ^1^Clinical Research Unit (CRU), Egas Moniz Center for Interdisciplinary Research (CiiEM), Egas Moniz School of Health & Science, Almada, Portugal; ^2^Aging Lab, Egas Moniz School of Health & Science, Egas Moniz Center for Interdisciplinary Research (CiiEM), Almada, Portugal; ^3^GENE - Artificial Nutrition Team - Gastroenterology Department - Hospital Garcia de Orta, Almada, Portugal

**Keywords:** institutionalized older adult, nursing home, nurse, caregiver, health care, oral health

## Abstract

**Introduction:**

This review aimed to evaluate the perceived barriers, knowledge, and training, of different health care professionals in relation to the oral health of dependent people in nursing homes and access, as well, how this data was evaluated.

**Methods:**

Three electronic databases—PubMed/MEDLINE, Web of Science, and LILACS—were searched independently by two researchers for relevant studies published up to December 2023. Articles were selected according to the established inclusion and exclusion criteria, and a total of 35 studies were included.

**Results:**

Findings from the Nursing Dental Coping Belief Scale studies revealed disparities between training and daily oral health care, with experienced nurses experiencing challenges. Barriers described were categorized and included resident-related issues, organizational challenges, and caregiver-related difficulties. Most caregivers reported inadequate training, often informal or experiential, although they are involved in the hygiene of the older adult, with tooth brushing and denture cleaning being the most common practices. Low oral health literacy coexisted with recognition of the importance of oral health care.

**Conclusion:**

The findings advocate for targeted interventions, standardized training, and improved support systems to improve oral health care for the older adult in diverse health care settings.

## 1 Introduction

The global shift to an older population continues to be one of the most significant societal changes of the 21st century, with the global population aged 65 years and older projected to exceed 1.5 billion by 2050 (1). As our population continues to age, the burden of chronic non-communicable diseases such as heart disease, cancer and musculoskeletal disorders will continue to increase (2). Oral diseases are no exception, and because they are often neglected, they continue to be a significant burden (3).

Tooth loss increases with age. According to 2017–2020 National Center for Health Statistics data, 13.2% of seniors have no natural teeth (4) Tooth loss can affect overall health and well-being. Edentulous older adults commonly experience compromised nutritional status, impaired speech function, and social discomfort, potentially leading to social isolation Seniors who have lost all of their teeth typically experience poor nutrition, difficulty speaking, and embarrassment, which can contribute to isolation (5). Nursing home residents, in particular, exhibit high rates of preventable or treatable oral/dental problems, including dental caries, gingivitis, periodontal disease, and gingival or oral discomfort and pain (6–9). The need to improve oral health care in nursing homes becomes even more urgent when we consider that the consequences of poor oral health are associated with an increased risk of malnutrition, aspiration pneumonia, respiratory disease, diabetes, and cardiovascular disease (10–12).

Health care professionals, such as nurses and aides, serve as the primary health care providers in nursing homes. Not only do they spend a considerable amount of time with the older adult, but they also have a significant impact on their health care (13). Although nurses recognize the importance of promoting oral health in frail older adults (14–16), the literature highlights the inadequacy of oral health education and training for health care professionals (17, 18). Unfortunately, dental health in older adults is often overlooked and remains an understudied area of research despite its importance in maintaining well-being, overall health, and quality of life (19, 20).

While there are scientific papers on oral health in nursing homes and institutionalized older adults, there are no systematic reviews on caregivers’ perceptions. This systematic review aims to evaluate caregivers’ perceptions of oral health care for dependent nursing home residents. The objectives were to summarize the methods used to assess barriers/difficulties, knowledge, training, available equipment, and perceptions of health care professionals regarding oral health care for dependent nursing home residents.

## 2 Methods

### 2.1 Protocol and registration

All authors drafted the protocol, registered it with the National Institute for Health Research PROSPERO (http://www.crd.york.ac.uk/PROSPERO, ID number: CRD42024497782), and reported it according to the Preferred Reporting Items for Systematic Reviews and Meta-Analysis (PRISMA) checklist (Supplementary Table S1).

### 2.2 Focused questions and eligibility criteria

We developed a protocol to answer the following PICO question: “What are the perceptions of health care professionals regarding oral health care for dependent nursing home residents?.” The respective statements were as follows:

- P (Participants): Health care professionals caring for dependent older adults in long-term care facilities.
- I (Intervention): No intervention was applied, as the focus was on health care professionals’ perceptions and practices.
- C (Control): The presence or absence of a control group was not a limitation.
- (Outcome): The outcome was the assessment of the perceptions, barriers, difficulties, knowledge, training, and available equipment for performing oral health care, as reported by health care professionals.

Cross-sectional observational studies were eligible for inclusion if they addressed the perceptions, difficulties, activities performed, and knowledge of health care professionals providing oral health care to dependent adults in long-term care facilities. Exclusion criteria were as follows: 1. duplicate studies; 2. abstracts, commentaries, reviews, letters to the editor, consensus, opinions, case studies, and case series; 3. unpublished information; 4. absence of the data being studied; 5. data obtained through a non-structured interview with non-comparable results; 6. population being family members as informal caregivers; and 7. articles written in languages other than English, Spanish, Portuguese, or French. There were no restrictions on the year of publication.

### 2.3 Data search strategy and study selection

We searched PubMed through PubMed/MEDLINE, Web of Science, and LILACS for all relevant articles published until December 2023. The following search terms were used: (1) (care home OR nursing home OR residential OR caregiver* OR care facilities); (2) (elder* OR senior* OR old OR aged OR geriatric); (3) (oral health OR oral care OR oral knowledge OR health care). Two independent reviewers (J.P.L. and I.R.) performed the search and included studies. Two independent reviewers independently assessed the titles and/or abstracts of the retrieved studies in duplicate (J.P.L. and I.R.), and disagreements were resolved by discussion with a third author (J.C.). For measurement reproducibility, inter-examiner reliability following full-text assessment was calculated using the kappa statistic.

### 2.4 Risk of bias assessment

The methodological quality of the eligible studies was assessed using the Newcastle-Ottawa Scale (NOS) (21), which was adapted for cross-sectional studies (Appendix 1). This adapted version of NOS evaluates three major domains for potential sources of bias: (1) selection bias (methods of participant selection), (2) comparability bias (methods of controlling for confounding variables), and (3) outcome bias (methods of assessing outcomes). Each of the seven items on the scale is assigned a star, with a maximum of one star per item. In this review, both selection bias and outcome bias were of particular concern due to the reliance on self-reported data, which can introduce a range of biases, such as recall bias or social desirability bias. Therefore, we assessed whether studies adequately controlled for such biases by using validated tools, objective measures, or triangulation of data sources where possible. The risk of bias assessment was conducted by two researchers (J.P.L. and I.R.), with any disagreements resolved by consulting a third researcher (J.C.). If a study was deemed to have a high risk of bias in any domain, we noted this in the quality assessment summary and took it into account when interpreting the findings.

### 2.5 Data extraction process and data items

Data extraction was performed independently by two reviewers (J.P.L. and I.R.), with discrepancies resolved through discussion with a third reviewer (J.C.). The following information was extracted from each eligible study: first author’s name, year of publication, country and location of sampling, sample size (male/female), mean age and mean years of experience, oral health perceptions of health care professionals, type of assessment, and study funding. For nurse perceptions, some specific information was collected from the studies for comparison: knowledge of dental terms/oral health; previous training to provide oral health care, type of training and perceived need for additional training; oral health care activities performed and availability of supplies to perform such care; access to oral health care by an oral health professional, perceived barriers/difficulties; and importance placed on oral health/relationship of oral health to systemic health.

We recognize that this review relied on self-reported data (e.g., surveys or interviews) to assess health care professionals’ perceptions and practices. While self-reported data are commonly used in research of this nature, they introduce a potential source of bias, such as social desirability bias, where respondents may report behaviors or attitudes, they believe are more socially acceptable or expected. Additionally, recall bias may influence the accuracy of self-reports, particularly when participants are asked to reflect on past experiences or behaviors. These limitations were considered when assessing the overall quality of the studies, and we critically discuss their potential impact on the findings in the subsequent sections.

For data analysis, standard spreadsheet software (Microsoft Excel for Mac, version 16.50. Microsoft, Redmond, WA, United States) was used to extract data. Frequencies and percentages were used to describe categorical variables, while continuous variables were reported as mean ± standard deviation (SD) and range.

## 3 Results

### 3.1 Study selection

**Figure 1:**
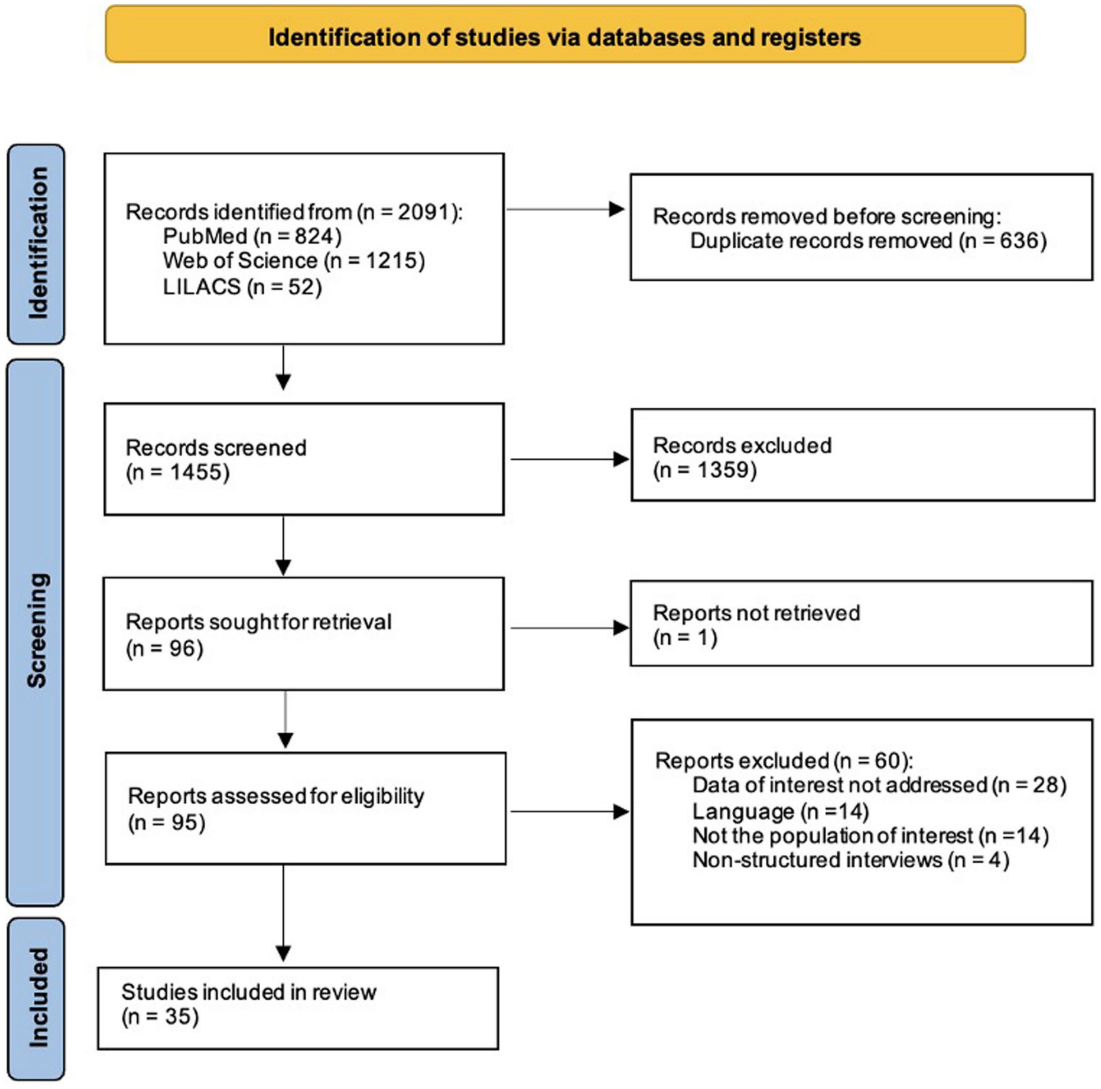
Prisma Flowchart.

The online search strategy identified 2,091 potentially relevant publications. After removing duplicates, 1,455 articles were assessed for eligibility criteria and 1,359 were excluded after title and/or abstract review. Of the 96 articles assessed for eligibility through full paper review, one could not be retrieved and 60 were excluded, with reasons for exclusion detailed in Supplementary Table S1. As a result, a final number of 35 observational studies were included for qualitative synthesis. The PRISMA plot is shown in Figure 1. The inter-observer reliability of the full-text screening was considered substantial (kappa score = 0.614, 95% CI: 0.471–0.757) (22).

### 3.2 Studies characteristics

A total of 6,179 participants, 4,219 women, and 554 men (1,406 did not report gender), from all 35 included studies were included in this systematic review (Table 1). The calculated percentage of 88.4% of female participants corroborates the literature, where most caregivers were female. The sample included personnel directly involved in providing oral health care to residents of health facilities: mostly nurses, assistant nurses, qualified aides, non-qualified aides, and some articles categorized them only as caregivers or careers (23–37). Others included diverse populations such as occupational therapists, speech therapists, social workers, physiotherapists, nursing students, dental nurses, and dental hygienists (15, 16, 38).

**Table 1:** Characteristics of the included studies.

Study	Country	Place of sample	Population	Sample (M/F)	Way of appraisal	Oral health care perceptions
						Case definition settings
Ballikaya et al. (23)	Turkey	Care home	Caregivers	147 (49/98)	Face-to-face questionnaire	1. Care practices of the disabled people; 2. Frequency of dental visits for the disabled people; 3. Dental plaque definition; 4. Effects of oral health in general health; 5. Attitude to oral care of the disabled people
Castillo et al. (51)	Brazil	Public geriatric care institution	Nurses, auxiliary nurses	24 (4/20)	Self-applied 14-item questionnaire based on previous studies	1. Working time in the institution; 2. Number of older adult people under their responsibility; 3. Previous training to carry out general and oral health care; 4. Availability of supplies for oral hygiene; 5. Tasks carried out in relation to oral hygiene care for the older adult; 6. Difficulties in carrying out such tasks
Catteau et al. (42)	France	8 Nursing homes (7 public and 1 private institution)	Nurses, auxiliary nurses, hospital agents	99 (3/96)	Self-applied 58-item questionnaire based on previous studies	1. Socio-demographic data and self-oral health attitudes; 2. Previously oral health training received, and interest in training opportunities; 3. Awareness of the relationship between oral disorders, systemic diseases and medical treatment; 4. 30 questions to assess oral health knowledge
Chebib et al. (41)	Switzerland	Trois-Chêne Hospital	Qualified nurses, nurses’ aides, care and community health assistants	NR	Online 39-item semi-structured questionnaire with some questions adapted from previous studies	1. General information on the caregiver; 2. Knowledge, clinical practices related to the oral care of dependent and independent inpatients and perceived importance of oral care in relation to general health; 3. Closed checklist of instruments; 4. Perception of oral care and potential barriers to its provision; 5. Perceived barriers concerning hospital logistics and patient-related factor
Cheng et al. (43)	Taiwan	7 Home-care facilities	Home-care aides (HAs)	312 (25/287)	Self-applied survey	1. Demographics of HAs; 2. Oral health care provision content: circumstances in which HAs provide oral health care to homebound patients and degree of comfort and knowledge that HAs have regarding their own skills in providing oral health care; 3. Views of HAs regarding providing oral health care services to patients
Chung et al. (39)	Switzerland	13 Nursing homes	Nurses, assistant nurses, qualified aides and non-qualified aids	169 (NR)	Self-applied questionnaire	1. Status, function, education, and number of years of experience of the nursing staff; 2. Attitude to their personal oral care and that of the residents; 3. Difficulties regarding oral hygiene care of the residents; 4. Responsibility for maintenance of residents’ oral hygiene; 5. Organization of professional dental care for the residents; 6. Major functions and responsibilities of a dentist caring for the residents
De Mello and Padilha (24)	Brazil	LTC facilities	Careers	36 (NR)	Semi-structured Interviews	1. Responsibility for oral care; 2. Oral care routines; 3. Difficulties in the conduction of oral care routines
Delgado et al. (52)	USA	3 Non-medical in-home care companies	Professional caregivers certified (CPC) and non-certified (NCPC)	67 (2/65)	NDCBS	1. Demographic information; 2. Close-ended oral care practice questions; 3. 4-part oral health belief Likert scale survey
Edman and Wårdh (50)	Sweden	Special accommodation sites; home care	Licensed nurse, assistant nurse, unit manager and other	2,167 (199/1933)	NDCBS and self-applied 28-item questionnaire	1. NDCBS; 2. Items covering background data: age, gender, number of years of experience, education, position at work, form of employment and workplace; 3. Supplementary questions about the importance of the resident’s oral health as well as the participants’ own oral health.
Forsell et al. (44)	Sweden	Geriatric nursing home	Nurses, nursing assistants, Nursing auxiliaries and Staff members with no formal education	87 (NR)	Questionnaire	1. Attitudes and perceptions among the nursing staff about providing the nursing home residents with daily oral care practices
Frenkel (25)	USA	22 Nursing homes	Careers	227 (NR)	Self-applied questionnaire with open-ended questions	1. Barriers to good oral health for clients; 2. Expressed need for training; 3. Suggested improvements in clients’ oral health care; 4. Carers’ comments on their own oral health
Garrido Urrutia et al. (26)	Chile	Long-term residence; local primary health domiciliary program	Formal caregivers from a long-term residence, informal caregivers from a local primary health domiciliary program	39 (1/38)	Structured questionnaires; NDCBS	1. Training in oral health care for dependent older adult; 2. Frequency of oral care practices; 3. Oral care practices; 4. Oral care practices related to denture use; 5. NDCBS
Godoy et al. (27)	Chile	Long-stay facilities	Caregivers	49 (0/49)	Interview + DCBS	1. Sociodemographic characteristics; 2. Oral care training; 3. Self-perceived need for training to perform oral care for an older adult person; 4. DCBS
Goh et al. (28)	Malasya	5 Nursing homes	Caregivers	96 (12/82)	24-item questionnaire	1. Demographics and job characteristics; 2. Attitudes and practices of the caregivers; 3. Exploratory analysis of the variables influencing the attitudes and PBC (Perceived Behavioral Control) scores of the caregivers
Hiltunen et al. (15)	Finland	LTC facilities	Nurse, practical nurse, nursing assistant, Nurse student, deaconess, dental nurse, occupational therapist, physiotherapist, elder care professional, mental health nurse, social worker, and homemaker	179 (6/150)	Self-applied 19-item questionnaire modified from 10-item questionnaire	1. Opinions and attitudes toward oral hygiene in older adults; 2. 19 statements/questions with five response alternatives and a space for free-hand comments on opinions and attitudes toward older adults’ daily oral hygiene
Ho et al. (14)	Malasya	15 nursing homes (NHs)—7 private and 8 voluntary welfare organizations (VWO)	Health care assistants, nursing aides, Enrolled nurses (ENs), registered nurses (RNs)	246 (67/179)	Self-applied online questionnaire adapted from previously published studies	1. Self-perceived oral health awareness; 2. Self-perceived oral health competence; 3. Perceived adequacy of geriatric oral care training received; 4. Impact of perceived adequacy of geriatric oral care training
Junges et al. (29)	Brazil	LTC institution	Permanent caregiver	24 (6/18)	Administered questionnaire based on a previous study	1. Orientation for general and oral hygiene and availability of materials for oral hygiene; 2. Frequency of difficulties in performing oral hygiene in relation to hygiene motivation in the independent older adult
Kohli et al. (45)	USA	8 LTC facilities	Certified nursing assistants, registered nurses, licensed practical nurse, unlicensed assistive support	70 (7/63)	Self-applied 21- item questionnaire was developed from the literature	1. Caregivers reported oral health training by perceived adequacy of training; 2. Perceived barriers/difficulties for carrying out oral health activities; 3. Caregivers’ expression of interest in oral health training ranked by most prioritized area
Paley et al. (17)	Australia	12 Facilities (high-level and low-level care)	Personal care assistants (PCAs); nursing position	40 (2/38)	Demographic questionnaire and semi-structured interview	1. Importance of oral health for residents; 2. Regularity of dental checkups for residents; 3. Oral health care activities in the facility; 4. Access to oral health care information; 5. Barriers to maintaining resident oral health; 6. Identification of needs or issues for residents of different ethnic backgrounds; 7. Suggestions for improvements of dental health services.
Palmers et al. (38)	Belgium	LTC settings	Nurse, nurse aid, head nurse, occupational therapist, speech therapist, social worker, physiotherapist, general practitioner and other	197 (21/176)	Online questionnaire based on an existing questionnaire	1. Personal characteristics; 2. Perception concerning the oral health of the residents, the perceived need for oral health education and skills training; 3. Caregivers’ profile in the organization, years of experience, and their role in daily oral care; 4. Perceived barriers and perception of current practices
Reis et al. (30)	Brazil	Public LTC facility for older adult people	Caregivers	27 (6/21)	Personal in-depth interviews and observation	1. Observation methods included analysis of caregiver’s emotions and routine of oral health care for the older adult; 2. Respondents’ education and professional experience; 3. Perceptions of the oral health of the institutionalized older adult; 4. Barriers or positive factors influencing their work
Rovida et al. (31)	Brazil	6 Nursing homes	Caregivers	42 (NR)	Semi-structured questionnaire	1. Concept of oral health; 2. Perception of the residents’ oral health conditions; 3. Suggestions on actions that could lead to an improvement in their dental condition
Sigurdardottir et al. (46)	Iceland	2 Nursing homes	Care assistants, practical nurses and registered nurses	107 (6/101)	NDCBS	1. NDCBS; 2. Socio-demographic and work experience; 3. Oral care activities equipment and dental supplies
Stančić et al. (32)	Serbia	4 Nursing homes	Caregivers	58 (5/53)	Self-administered questionnaire	1. Main barriers to maintaining oral hygiene of the residents; 2. Procedures that the caregivers apply to maintain oral hygiene of the residents; 3. Caregivers’ knowledge of symptoms and prevention of caries and periodontal disease
Sumi et al. (16)	Japan	Nursing homes	Nurses, nursing home caregivers, nutritionists, dental hygienist and other staff caregivers	410 (84/326)	Self-report questionnaires	1. Awareness of oral care; 2. Training in oral care; 3. Burden of oral care; 4. Systematization of oral care
Unfer et al. (33)	Brazil	7 Long-stay institutions (5 private and 2 public)	Caregivers	26 (1/25)	Semi-structured interviews conducted by 2 researchers	1. Perceptions about the oral health of older adult; 2. Procedures of oral care in older adult in the institution; 3. Characteristics of the oral care of older adult in the institution and the caregiver’s competence and need for training in this area; 4. Procedures of the self-oral care of the caregiver
Vieira et al. (34)	Brazil	5 Geriatric institutions	Caregivers	59 (16/43)	Structured interview conducted by 3 researchers	1. Participant identification data; 2. Data related to knowledge about oral diseases and their prevention methods, aspects related to the cleaning of dentures, oral health care and the involvement of caregivers in the oral health of the older adult; 3. Interest in receiving information about oral health care and what information is of greatest interest
Villarosa et al. (35)	Australia	Dependent living units, nursing homes and dementia-specific care	Care staff	12 (1/11)	Semi-structured interviews	1. Significance of oral health in residential aged care; 2. Care staff as primary oral hygiene providers; 3. Challenges in providing oral health care; Strategies to improve oral health care
Wårdh et al. (47)	Sweden	12 nursing homes	Nursing home personnel	454 (NR)	Self-applied questionnaire	1. Demographic; 2. 16 multiple-choice questions on attitudes to and knowledge about oral health care; 3. Open item for free comments about oral health care work
Wårdh et al. (40)	Sweden	3 service houses/centers for home care, 2 nursing homes and 2 apartment homes for the demented older adult	8 nursing assistants and 14 home-care aids	22 (2/20)	Semi-structured interviews	Subject’s own description of assisting oral health care, thoughts, feelings and actions in the situations described
Webb et al. (48)	Australia	Aged care facilities	Directors of Nursing (DON)	NR	Self-applied questionnaire	1. Dental care; 2. Oral health status of residents; 3. DON/staff opinions
Webb et al. (36)	Australia	Aged care facilities	Careers	211 (NR)	Mailing self-applied 23-item questionnaire	1. Career demographics; 2. Oral care in the ACF; 3. Resident’s oral care; 4. Factors that influence oral care
Weening-Verbree et al. (18)	Netherlands	24 nursing home	32 registered nurses, 365 nurse assistants and nurse aids and 12 nursing home managers	409 (NR)	Self-applied questionnaire	1. Background information nursing staff; 2. Oral status of residents; 3. Performance of oral care in residents
Wilk et al. (37)	USA	Home health agencies	Caregivers	69 (10/59)	Mailing self-applied 39-item questionnaire	1. Caregiver demographics; 2. Caregiver’s personal oral health behaviors; 3. Oral health to a client; 4. Health Literacy in Dentistry (HeLD)-14
Willumsen et al. (49)	Norway	Nursing home	Registered nurses, auxiliary nurses and assistant nurses	494 (19/430)	Self-applied questionnaire	1. Resistance to tooth cleaning; 2. Results from nurses’ (N) attitudes (A), implementation (I) and knowledge (K)—NAIK—questionnaire: 4 items about attitudes to oral health, 6 items about implementation opportunities, and 6 items about the importance of knowledge

LTC, long term care; NR, Not reported; M, Male; F, Female; DCBS, Dental Coping Beliefs Scale.

Of the 35 articles included, 9 articles (about 17%) were published before 2010 (16, 17, 24, 25, 39, 40), with the oldest article published in 1999. All the remaining articles were published after 2010, and about 43% of them were published after 2015. The latest article was published in 2023.

Most of the studies were conducted in care facilities for the older adult, with the exception of one study developed in a hospital setting (41).

Several issues were considered in the case definition setting. Some studies addressed more than one issue: 29 studies searched barriers or difficulties felt when performing oral health care activities (14–18, 23–25, 28–33, 35–49), 13 studies assessed the perceived importance of oral health care (28, 30, 31, 34, 35, 41, 42, 50, 14–18), 19 accessed oral health knowledge (14, 18, 23, 28, 30–37, 40, 42, 46–49, 51), 20 studies emphasized previous training received (14–16, 18, 23, 26–28, 32, 35, 39–43, 46–48, 51, 52), and 24 the perceived need for training (14–18, 23, 25, 27, 30, 32–38, 40, 42, 45–50). The other 14 studies mentioned the access to oral health care by an oral health professional (14–18, 24, 30, 31, 35–37, 39, 40, 42, 48), 22 studies explored the oral health care activities performed (14, 18, 23, 24, 26, 29–37, 40, 41, 43, 46–49, 52), and 7 studies evaluate if supplies to perform such oral health care activities were available (18, 25, 29, 30, 32, 33, 46).

The methods used to collect data on oral health care provided by caregivers varied between studies and some applied more than one. Questionnaires were used in 24 studies (14–16, 18, 23, 25, 26, 28, 29, 31, 32, 36–39, 41, 42, 44, 45, 47–49, 51), semi-structured interviews in 8 studies (17, 24, 27, 30, 33–35, 40), and a more systematic data collection approach using the NDCBS in 5 studies (26, 27, 46, 50, 52).

Furthermore, studies were conducted in 17 countries worldwide: Turkey (23), Brazil (24, 29–31, 33, 34, 51), France (42), Switzerland (39, 41), Taiwan (43), USA (25, 37, 45, 52, 53), Sweden (40, 44, 47, 50), Chile (26, 27), Malaysia (14, 28), Finland (15), Australia (17, 35, 36, 48), Iceland (46), Serbia (32), Japan (16), Netherlands (18), Norway (49) and Belgium (38). Of note, no studies were performed in Africa.

### 3.3 Methodological quality of the included studies

The methodological quality of the studies varied significantly, with most studies falling into the fair (31.4%, *n* = 11) or high (28.6%, *n* = 10) quality categories, and 7 studies rated as low quality (Table 2). None of the included studies described and calculated the non-response rate (item 3). Studies mostly failed to identify confounding factors and to perform a subgroup or multivariable analysis taking them into account (51.4%, *n* = 18) (item 5) and to use a validated screening/measurement tool (88.6%, *n* = 31) (item 4). This presents a concern regarding the reliability and generalizability of the findings.

**Table 2:** Results from the methodological appraisal using Newcastle-Ottawa Quality Assessment Scale adapted for cross sectional studies.

Study	1	2	3	4	5	6	7	Overall
Ballikaya et al. (23)	1	1	0	1	0	2	1	Fair
Castillo et al. (51)	1	1	0	1	1	2	1	High
Catteau et al. (42)	1	1	0	1	0	2	1	Fair
Chebib et al. (41)	1	1	0	1	0	2	1	Fair
Cheng et al. (43)	1	0	0	1	0	2	1	Fair
Chung et al. (39)	1	1	0	1	1	2	1	High
De Mello and Padilha (24)	1	1	0	2	0	1	0	Fair
Delgado et al. (52)	1	0	0	0	0	2	0	Low
Edman and Wårdh (50)	1	1	0	2	0	2	1	High
Forsell et al. (44)	1	0	0	1	1	2	1	Fair
Frenkel (25)	1	1	0	1	0	2	0	Fair
Garrido Urrutia et al. (26)	1	1	0	2	1	2	1	High
Godoy et al. (27)	1	1	0	2	0	2	1	High
Goh et al. (28)	1	1	0	1	1	2	1	High
Hiltunen et al. (15)	1	1	0	1	0	2	1	Fair
Ho et al. (14)	1	1	0	1	1	2	1	High
Junges et al. (29)	1	0	0	1	0	2	1	Fair
Kohli et al. (45)	1	1	0	1	1	2	1	High
Paley et al. (17)	1	1	0	0	0	1	1	Low
Palmers et al. (38)	1	1	0	1	1	2	1	High
Reis et al. (30)	1	1	0	0	0	1	1	Low
Rovida et al. (31)	1	1	0	1	0	2	1	Fair
Sigurdardottir et al. (46)	1	1	0	1	1	2	1	High
Stančić et al. (32)	1	1	0	1	1	2	1	High
Sumi et al. (16)	1	1	0	1	0	2	1	Fair
Unfer et al. (33)	1	1	0	0	0	1	1	Low
Vieira et al. (34)	1	1	0	0	0	1	1	Low
Villarosa et al. (35)	1	1	0	0	0	1	1	Low
Wårdh et al. (47)	1	1	0	1	0	2	1	Fair
Wårdh et al. (40)	1	1	0	0	0	1	1	Low
Webb et al. (48)	1	1	0	1	0	2	1	Fair
Webb et al. (36)	1	1	0	1	0	2	1	Fair
Weening-Verbree et al. (18)	1	1	0	1	0	2	1	Fair
Wilk et al. (37)	1	1	0	1	0	2	0	Fair
Willumsen et al. (49)	1	1	0	1	0	2	1	Fair

Item 1—Representativeness of the cases; Item 2—Sample size; Item 3—Non-Response rate; Item 4—Ascertainment of the screening; Item 5—The potential confounders were investigated by subgroup analysis or multivariable analysis; Item 6—Assessment of the outcome; Item 7—Statistical test; Overall—Sum of the value obtained in each item.

The heterogeneity of the studies was also evident, as different data collection methods were used, including questionnaires (24 studies), semi-structured interviews (8 studies), and the Nursing Dental Coping Belief Scale (NDCBS) (5 studies). This methodological diversity complicates direct comparisons between studies and highlights the potential for bias introduced by the lack of standardization in measurement tools. Furthermore, the study populations varied widely, including different categories of health care professionals (e.g., nurses, aides, dental professionals) across various countries, settings (nursing homes vs. hospitals), and types of training (formal vs. informal). This variation in study design and execution calls for caution in interpreting the aggregated results and underscores the need for more standardized approaches in future research.

### 3.4 Synthesis of evidence

#### 3.4.1 Nursing dental coping belief scale

The Nursing Dental Coping Belief Scale (NDCBS), originally validated in the U.S. for male veterans (54), was adapted for use with health care professionals in nursing settings (55). The aim was to create an oral health care priority index that could be used in both hospital wards and specialized facilities. The instrument consists of a 28-item questionnaire covering four dimensions: internal locus of control (IL), external locus of control (EL), self-efficacy (SE), and oral health care beliefs (OHCB). Lower scores represent an individual’s positive DCB and strong belief in their ability and competence to influence oral health behaviors. Four of the included studies (26, 27, 46, 52) used the NDCBS. The scale measures four dimensions: internal locus of control, external locus of control, self-efficacy, and beliefs about oral health care. Studies using the NDCBS have found that nurses’ beliefs about their ability to influence oral health behavior were often overly optimistic, with many overestimating their knowledge and skills. However, their actual practice did not always support this self-assessment bias (26, 52). In some studies, nurses with more formal training showed better beliefs about their competence (27, 46). In constrast, while other studies showed that more extended work experience was paradoxically associated with poorer dental coping beliefs (46). The inconsistency of these findings points to the heterogeneity of carers’ perceptions, which personal attitudes, educational background, and workplace dynamics may influence.

#### 3.4.2 Perceived oral care barriers

The barriers or difficulties experienced by caregivers in providing oral health care to residents, which were mentioned in 29 of the included studies (14–18, 23–25, 28–33, 35–49), were categorized into 3 groups: barriers related to the residents themselves, barriers related to the organization, and barriers related to the caregiver (Table 3).

**Table 3:** Barriers/difficulties perceived by the caregivers and mentioned in the included studies (*n* total = 29 studies).

	Studies (*n*)
Related to the residents themselves	
Non-cooperative/ challenging behavior	15
Lack of interest/motivation	3
Presenting with critical medical conditions/ debility	2
Refusal of oral care	2
Organization related	
Lack of time to provide oral health care	17
Insufficient materials/ lack of resources	6
Lack of staff	5
Lack of regular on-site support from dental professionals	2
Related to caregiver	
Inadequate education/skills to provide oral health care	9
Not liking the procedure because of nausea/ feeling of disgust	4
Fear of causing damage	3
Lack of prioritization	2

*n, number of studies.*

In terms of barriers related to the residents themselves, lack of cooperation was the most frequently reported, in 15 studies (14, 18, 24, 25, 28–30, 32, 35, 39, 41, 44, 45, 47, 48). Negative attitudes, bad moods, cursing, and even physical violence are some of the challenging behaviors exhibited by the residents and reported by caregivers. Other barriers include residents’ lack of interest or motivation (32, 41, 44), residents’ critical illness or debility (30, 41), and residents’ refusal of oral health care (41, 45).

Most caregivers report that they do not have time to provide oral hygiene to the residents (15, 18, 24, 25, 28, 29, 32, 33, 38–43, 45–47). Lack of oral hygiene materials (18, 28, 32, 38, 41, 48), lack of staff (33, 38, 41, 42, 47), and lack of regular on-site support from dental health professionals (14, 35) are also reported as organizational barriers. Caregivers also report not having adequate training or skills to provide oral health care (28, 31, 35, 37–40, 46, 48). In addition, motives such as disgust or lack of association with the procedure (15, 23, 25, 39), fear of causing harm (25, 28, 44), or lack of prioritization (18, 35) have also been reported as caregiver-related difficulties in providing oral health care.

The variability in the nature and extent of these barriers across studies highlights the heterogeneity of care contexts and the complexity of addressing these challenges.

#### 3.4.3 Training in providing oral health care

Table 4 shows the number and percentage of caregivers who received training in oral health care and the type of training received. In most studies, less than half of the caregivers reported receiving training in oral health care for the older adult (16, 18, 23, 26–28, 39, 42, 43). Unfortunately, not all of these studies evaluated the type of training received. Those that did so concluded that, in most cases, the training was informal or based on personal experience (28, 32, 42, 43, 47, 51). However, in almost all studies that assessed the need for training, participants were interested in implementing training programs (14–16, 18, 23, 27, 32, 35, 40, 42, 46–48). This gap between the need for training and the actual provision of training reflects an important organizational barrier. It highlights the potential for improving nurse education to improve oral health care practice.

**Table 4:** Received previous training and type of training received.

Study	Training received - n (%)	Type of training
Ballikaya et al. (23)	52 (35.4)	NR
Castillo et al. (51)	NR (43.9)	Training course by the staff members of the institutions to help new arrivals.
	NR (24.4)	At school by a mentor.
Catteau et al. (42)	91 (45)	Unspecified practical training in oral hygiene (given by the staff members of the institutions assisting new arrivals or by a mentor in the school).
Cheng et al. (43)	164 (48.2)	The facilities employ instructors for training.
	76 (22.4)	Self-learning during employment.
	38 (11.2)	Seniors provide guidance.
	39 (11.5)	Consulting dentists and other professionals.
	23 (6.8)	Other.
Chung et al. (39)	NR (31)	NR
Delgado et al. (52)	NR (60)	NR
Garrido Urrutia et al. (26)	17 (43.6)	NR
Godoy et al. (27)	18 (36.73)	NR
Goh et al. (28)	14 (15)	From institution.
	28 (30)	Only on-the-job training.
	40 (43)	Both institutional and on-the-job.
Hiltunen et al. (15)	118 (65.9)	NR
Ho et al. (14)	NR (68.1)	NR
Stančić et al. (32)	NR (81)	Most caregivers had learned oral hygiene techniques from colleagues (41.4%)
Sumi et al. (16)	NR (39)	NR
Wårdh et al. (47)	NR (65)	Formal training in oral health as part of their basic education and/or during their employment.
Wårdh et al. (40)	NR	Postgraduate training.
Webb et al. (48)	NR (74.7)	66.7% had undertaken formal training of which 50.1% was in-house.
Weening-Verbree et al. (18)	NR (43)	The frequency, content, and duration of education about oral health education was unclear from the responses.

*n, number of caregivers; NR, not reported.*

#### 3.4.4 Oral health knowledge, importance given to oral health, and oral health care activities performed

A total of 18 studies (14, 18, 23, 28, 30–37, 40, 42, 46–49, 51) assessed oral health knowledge using different measures. However, the conclusions were consistent with low oral health knowledge. Gaps in oral health knowledge include beliefs that tooth loss is an inevitable part of aging (14, 28) or that caries is a communicable disease, and lack of information about periodontitis (32, 34, 42). In a single study (51), caregivers were highly educated in the theoretical context, but this wasn’t reflected in the oral hygiene of the older adult as observed by the mucosal and plaque index. Although oral health literacy is low, participants recognize the importance of providing oral health care to residents and are aware of the interaction of systemic diseases and medical treatments with oral disease and the well-being of the older adult (14–18, 28, 30, 31, 34, 35, 41, 42, 50).

A total of 22 studies reported oral health activities performed by caregivers (14, 18, 23, 24, 26, 29–37, 40, 41, 43, 46–49, 52). The most common performed oral hygiene activity was tooth brushing (14, 18, 23, 24, 26, 29–37, 40, 41, 43, 46–49, 52) followed by denture cleaning (14, 18, 24, 26, 29–33, 35–37, 41, 43, 46, 48, 49). Other activities such as rinsing the mouth with a mouthwash (24, 26, 32, 35, 43), removing dentures for sleep (26, 41), cleaning the oral mucosa with a gauze in the absence of teeth (26, 29, 30), and flossing (26, 36) were also performed, although with a much lower frequency. While some caregivers confirmed that the necessary materials to provide oral health care were available in the facilities (29, 30, 33, 46), others expressed concern about the lack of resources, such as toothbrushes (18, 25, 32). The heterogeneity of practice across studies and settings further complicates the interpretation of findings, as some studies reported caregivers performing multiple oral health tasks. In contrast, others focused primarily on brushing or denture care.

Access to oral health care by an oral health professional was assessed in 14 of the included studies (14, 15, 17, 18, 24, 30, 31, 35–37, 39, 40, 42). Most staff support the availability of dental chairs or an on-site dentist with portable dental units and regular visits by oral health professionals (31, 35, 37, 39). However, home visits are not followed up and regular check-ups in nursing homes are rare (17, 40). Access to emergency care is a challenge, with reliance on local dentists and delays (36, 48). Only one study mentioned regular oral health campaigns, where a dentist goes to the home care facility to examine the older adult (30). These variations highlight the contextual of care provision and the need for more robust infrastructure and support for carers in many settings.

## 4 Discussion

### 4.1 Summary of main findings

This systematic review provides an in-depth analysis of the oral health care challenges that carers of dependent older adults, face. It highlights several key issues: the gap between education and practice, the persistence of barriers to adequate oral health care, and lack of health literacy among carers. The reviewed studies show that although carers recognize the importance of oral health and its link to systemic health, their ability to provide adequate care is often troubled by insufficient formal training, inadequate resources, and organizational challenges. Caregivers were primarily involved in brushing teeth and cleaning dentures but were less likely to perform more complex oral health tasks. Furthermore, despite these challenges, carers demonstrated a strong awareness of the need for oral care in older people, although their knowledge of oral health practices and conditions remained limited.

Results from studies using the Nursing Dental Coping Belief Scale (NDCBS) show a significant discrepancy between carers’ beliefs about their competence to provide oral care and the actual practices observed. Experienced carers often reported facing more challenges, possibly due to burnout or a mismatch between training and the demands of caring. The barriers identified across studies can be categorized into resident, organizational, and carer-related factors, each contributing to suboptimal oral health care.

### 4.2 Implications for practice and research

The included studies showed that oral health care practices for dependent older adults are still inadequate, insufficient, and unsystematic. Although guidelines for appropriate oral health care exist (56, 57), training in oral and prosthetic hygiene has been shown to have a positive impact, and various oral health training programs for care providers working in geriatric settings have been described in the literature (58–62). However, a systematic review of strategies to improve oral health care showed that there is still a need to improve the strategies used to change oral health care behaviors, as providing general information seems to be successful in increasing oral health knowledge but does not necessarily improve oral health (63). In addition, another systematic review (64) showed that oral health education programs may indeed have a positive effect on oral hygiene in the older adult, although some limitations of the included studies were noted.

Therefore, caregivers need structured training programs that improve their knowledge and equip them with the skills and resources to effectively perform daily oral health tasks. Training programs can be more effective if they are tailored to the specific needs of caregivers in different settings and focus on practical training. In addition, such training should be regularly updated to reflect advances in oral health care for older people and integrated into the routine activities of nursing homes and care facilities. Dental professionals must actively participate in training and provide ongoing support, as this significantly improves caregivers’ confidence and competence in delivering oral health care.

In addition, the financial burden of dental care for nursing home residents remains a significant issue. Oral health care is often excluded from public health coverage, leaving residents to pay for treatment. This factor contributes to the neglect of oral health and increases the risk of significant oral disease. We must implement policy changes to integrate dental care into the broader health care framework for older people and provide financial support to reduce out-of-pocket costs for residents.

### 4.3 Strengths and limitations

This systematic review was conducted according to PRISMA, a rigorous and widely recommended guideline that increases robustness and reduces reporting errors. In addition, an extensive literature search was conducted using a meticulous predefined protocol.

However, there are some limitations that need to be discussed. Most studies used a convenience sample of nursing homes in the study area, so the results may have been different if the other facilities had been included in the studies. In addition, only a few health care professionals from each sample site participated in the surveys. As a result, the small sample size limits the ability to extrapolate the data to the rest of the population and the ability to detect small differences between groups as statistically significant. Another limitation is the reliance on self-reported data, particularly from questionnaires and interviews, which can introduce various forms of bias. Carers may be motivated to give socially desirable answers, overestimating their level of training or the quality of care they provide. Recall bias is also a concern, as caregivers may have difficulty accurately recalling specific events or practices related to oral health care. In addition, the heterogeneity of the studies—ranging from differences in data collection methods (e.g., questionnaires vs. interviews) to differences in study populations (e.g., type of caregiver, setting, geographic location)—makes it difficult to draw firm conclusions about the generalisability of the findings. The lack of standardized measurement tools across studies makes it difficult to compare results, especially for complex constructs such as oral health knowledge and caregiver self-efficacy.

Future studies should focus on data representativeness and method standardization to ensure more homogeneous evidence-based results. The NDCBS is a standardized assessment tool that should be widely used. This information is extremely important for improving the oral health of nursing home residents and, consequently, their well-being and systemic health. It is also important for educating nursing home administrators about the improvements that can be made in oral health care.

### 4.4 Recommendations for overcoming barriers

The findings of this review support the proposal of several actionable strategies to address the barriers to providing oral health care for older adults:

1. Standardize training programs: Institutions can formalize nursing training, incorporating hands-on sessions that focus on practical aspects of oral health care, especially for non-dental professionals. These programs should be integrated into nurses’ induction processes and continuing education initiatives, ensuring they acquire and maintain up-to-date knowledge and skills.
2. Improve access to resources: Facilities can ensure the availability of adequate oral health supplies, including toothbrushes, denture care products, and other essential materials. Regular efforts are needed to maintain the accessibility and readiness of these resources for staff use.
3. Policy changes for financial support: Governments and health systems can extend dental care coverage for older people in long-term care facilities. This may involve incorporating dental services into existing health programs or creating separate funding for dental care for the older adult.
4. Regular monitoring and support: Ongoing support from dental professionals should be integrated into the care routine for older residents, ensuring that carers have access to advice when needed. In addition, regular monitoring of oral health outcomes should be implemented to identify problems early and improve the overall quality of care.

## 5 Conclusion

This review highlights the multiple barriers to oral health care for dependent older adults, including time constraints, lack of training, inadequate resources, and poor collaboration among caregivers. In particular, caregiver training programs are often informal and experiential, while oral health literacy remains low, creating a critical gap in their ability to provide adequate care. The included studies’ methodological limitations, such as reliance on self-reported data and lack of standardized measures, highlight the need for more robust and standardized research designs.

To address these challenges does not appear to be modifying the subject structured, evidence-based training programs for caregivers. These programs should be comprehensive, combine theoretical knowledge with practical skills, and directly address the barriers identified in this review. In addition, systemic changes are needed to ensure that older adult residents have financial access to dental care, often a significant barrier to optimal care.

Future research should focus on overcoming the limitations of current studies by standardizing data collection methods and using validated instruments, such as the NDCBS, to ensure greater comparability between studies. Longitudinal studies or randomized controlled trials are essential to assess the effectiveness of different educational programs and interventions in improving oral health knowledge and clinical outcomes in older populations.

Researchers must investigate the cost-effectiveness of integrating oral health care into long-term care and develop strategies to incentivize dental professionals to participate in routine care. They should also analyze the benefits of interdisciplinary care models that include nurses and dental professionals and evaluate how policy changes can improve access to dental care for older adults, especially in regions with limited public dental coverage.

## Data Availability

The original contributions presented in the study are included in the article/Supplementary material, further inquiries can be directed to the corresponding author.
