# Supplementary material for: Health professionals’ perceptions, barriers and knowledge towards oral health care of dependent people in nursing homes: a systematic review
Source: Front Public Health. 2025 Jan 23;12:1504542. doi: 10.3389/fpubh.2024.1504542 (PMC11798777; doi:10.3389/fpubh.2024.1504542)
Supplement: Supplementary file 1 [file Supplementary_file_1.docx]

Supplementary Material

**Supplementary Table S1.** Excluded articles with reason

| **Reference** | **Year** | **Reason for exclusion** |
| --- | --- | --- |
| Adebayo et al. | 2017 | Not the population of interest: African migrant carers’ |
| Blanco et al. | 1997 | Data of interest not addressed |
| Blank et al. | 1996 | Data of interest not addressed |
| Bonfá et al. | 2017 | Not the population of interest: informal caregivers |
| Chen et al. | 2023 | Data of interest not addressed |
| Chiba et al. | 2008 | Data of interest not addressed |
| Cho et al. | 2013 | Language: chinese |
| Coleman and Watson | 2006 | Data of interest not addressed: Oral Care Provided by Certified Nursing |
| Coleman, P | 2005 | Data of interest not addressed: Evaluation of the effect of oral health care administered |
| Cornejo-Ovalle et al. | 2013 | Data of interest not addressed |
| Dharamsi et al. | 2009 | Data of interest not addressed: Assessment of the implementation of a education program |
| Eadie and Schou | 1992 | Non-structured interviews with open-ended questions and non-comparable results |
| Ezenwa et al. | 2016 | Not the population of interest: residents are specificly cancer patients |
| Forsell et al. | 2010 | Data of interest not addressed: Impact of an oral hygiene educational program |
| Girestam Croonquist C | 2020 | Data of interest not addressed: Evaluation of the effect of oral health care administered |
| Godoy & Rey et al. | 2019 | Data of interest not addressed |
| Gomez-Rossi et al. | 2022 | Data of interest not addressed |
| Gopalakrishnan et al. | 2019 | Not the population of interest: Assessment of home care manager's perception about oral care |
| Gu et al. | 2023 | Data of interest not addressed |
| Ho et al. | 2023 | Not the population of interest: dental therapists |
| Holmavuo et al. | 2022 | Not the population of interest: informal caregivers |
| HONG et al. | 2015 | Language: chinese |
| Idris et al. | 2023 | Data of interest not addressed: Comparison of caregivers oral care of the elderly between care homes and self-home |
| il et al. | 2017 | Language: chinese |
| Jeon et al. | 2015 | Language: chinese |
| Jinah et al. | 2021 | Language: chinese |
| Jones et al. | 2019 | Not the population of interest: Family members and caregivers |
| Kalsbeek et al. | 2006 | Language: dutch |
| Kang | 2016 | Language: chinese |
| Koistinen et al. | 2019 | Data of interest not addressed |
| Kyung et al | 2014 | Language: chinese |
| Lee et al. | 2013 | Language: chinese |
| Letchumanan et al. | 2020 | Data of interest not addressed: Assessment of health care provided by external entities |
| Linquist et al. | 2012 | Non-structured interviews with open-ended questions and non-comparable results |
| Maramaldi et al. | 2019 | Data of interest not addressed |
| Marques et al. | 2016 | Data of interest not addressed |
| Matear and Barbaro | 2006 | Not the population of interest: Family members and caregivers |
| Mckelvey et al. | 2003 | Non-structured interviews with open-ended questions and non-comparable results |
| Mello et al. | 2010 | Not the population of interest: dental surgeons |
| Minihan et al. | 2014 | Not the population of interest: adults with developmental disabilities |
| Mo and Kim | 2008 | Language: chinese |
| Ohara et al. | 2021 | Not the population of interest: informal caregivers |
| Paley et al. | 2009 | Not the population of interest: residents and family overview |
| Park and Choi-Kwon | 2011 | Language: corean |
| Park et al. | 2022 | Language: chinese |
| Patterson Norrie et al. | 2019 | Non-structured interviews with open-ended questions and non-comparable results |
| Preston et al. | 2006 | Data of interest not addressed |
| Reed et al. | 2006 | Data of interest not addressed |
| Reznick & Matear | 2015 | Data of interest not addressed |
| Schembri et al. | 2005 | Not the population of interest: home care managers |
| Sjögren et al. | 2010 | Data of interest not addressed: Assessment of elderly's oral health |
| Thean et al. | 2007 | Data of interest not addressed |
| Urata et al. | 2018 | Not the population of interest: Nursing home managers |
| Vanobbergen et al. | 2005 | Data of interest not addressed |
| Veerasamy et al. | 2023 | Data of interest not addressed: Assessment of caregiver's current education |
| Wiener et al. | 2014 | Data of interest not addressed |
| Yoon et al. | 2020 | Data of interest not addressed |
| Young et al. | 2008 | Data of interest not addressed: Assessment of caregiver's knowledge before and after oral care education |
| 김기욱; 김지화 | 2015 | Language: chinese |
| 송설경; 성정민 | 2014 | Language: chinese |
| Pyle et al. | 1999 | Data of interest not addressed |

**Appendix 1.** Newcastle-Ottawa Scale adapted for cross-sectional studies

​

**Selection: (Maximum 5 stars)**

1. Representativeness of the sample:

a. Truly representative of the average in the target population. * (all subjects or random sampling)

b. Somewhat representative of the average in the target group. * (non-random sampling)

c. Selected group of users/convenience sample.

d. No description of the derivation of the included subjects.

2. Sample size:

a. Justified and satisfactory (including sample size calculation). *

b. Not justified.

c. No information provided

3. Non-respondents:

a. Proportion of target sample recruited attains pre-specified target or basic summary of non-respondent characteristics in sampling frame recorded. *

b. Unsatisfactory recruitment rate, no summary data on non-respondents.

c. No information provided

4. Ascertainment of the exposure (risk factor):

1. Validated measurement tool. **
2. Non-validated measurement tool, but questionnaire is presented. *
3. No description of the measurement tool

**Comparability: (Maximum 2 stars)**

1. Comparability of subjects in different outcome groups on the basis of design or analysis. Confounding factors controlled.

a. Data/ results adjusted for relevant predictors/risk factors/confounders e.g. age, sex, work experience, training, etc. **

b. Data/results not adjusted for all relevant confounders/risk factors/information not provided.

**Outcome: (Maximum 3 stars)**

1. Assessment of outcome:

1. Structured questionnaire**
2. Semi-structured or structured interview*
3. Reported by other
4. No description

2. Statistical test:

a. Statistical test used to analyse the data clearly described, appropriate and measures of association presented including confidence intervals and probability level (p value). *

b. Statistical test not appropriate, not described or incomplete.

Thresholds for converting the Newcastle-Ottawa scales to AHRQ standards (good, fair, and poor):

- **Good quality:** 7-10 points
- **Fair quality:** 5/6 points
- **Poor quality:** less than 5 points

This scale has been adapted from the Newcastle-Ottawa Quality Assessment Scale for cohort studies to provide quality assessment of cross-sectional studies
